# Patterns of testosterone in male white‐tailed deer (*Odocoileus virginianus*): Seasonal and lifetime variation

**DOI:** 10.1002/ece3.7423

**Published:** 2021-03-21

**Authors:** Monet A. Gomes, Stephen S. Ditchkoff, Sarah Zohdy, William D. Gulsby, Chad H. Newbolt

**Affiliations:** ^1^ School of Forestry and Wildlife Sciences Auburn University Auburn Alabama USA; ^2^ Department of Pathobiology College of Veterinary Medicine Auburn University Auburn Alabama USA

**Keywords:** androgens, reproductive ecology, seasonality, testosterone, ungulates

## Abstract

Testosterone is strongly associated with the annual development of antlers in cervids, but endocrine research on wild, freely breeding ungulates is often done without repeated capture of known‐aged individuals. As a result, our knowledge on how testosterone fluctuates over the course of a lifetime and variation in lifetime patterns among individuals is limited. We investigated patterns of testosterone in a freely breeding population of white‐tailed deer (*Odocoileus virginianus*) in Alabama, USA, that breeds in January. Testosterone peaked during the height of the breeding season, despite this period occurring approximately 2 months later than in most temperate‐region white‐tailed deer populations. Age‐related differences in testosterone were only prevalent during the breeding season, with bucks ≥3.5 years old having greater testosterone (853 ng/dl ± 96 *SE*; *p* = 0.012) than bucks 1.5–2.5 years old (364 ng/dl ± 100 *SE*). Additionally, an individual's testosterone level as a yearling was not positively associated with their lifetime maximum testosterone level (*p* = 0.583), and an individual's mean testosterone level was positively associated with lifetime testosterone variation (*p* < 0.001). To our knowledge, our study is one of the first to assess how testosterone early in life might relate to individual testosterone later in life. We believe these data provide insight into lifetime hormonal patterns in cervids, and that these patterns may indicate intraspecific variation of lifetime reproductive strategies.

## Graphical abstract

We investigated patterns of testosterone in a freely breeding population of white‐tailed deer (*Odocoileus virginianus*) in Alabama with a January breeding period. Testosterone peaked during the height of the breeding season, and age‐related differences in testosterone were only prevalent during this time, with bucks ≥3.5 years old having greater testosterone (853 ng/dl ± 96 *SE*; *p* = 0.012) than bucks 1.5–2.5 years old (364 ng/dl ± 100 *SE*). Additionally, an individual's testosterone level as a yearling was positively associated with their lifetime maximum testosterone level (*p* = 0.006), and an individual's mean testosterone level was positively associated with lifetime testosterone variation (*p* < 0.001).

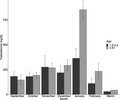

## 1 INTRODUCTION

In many vertebrates, testosterone has been linked to dominance behaviors associated with copulation and reproduction (Knol & Egberink‐Alink, 1989; Rose et al., 1971). For members of the family Cervidae, testosterone plays a vital role in antler development and facilitates the behaviors and development of physical characteristics necessary for breeding (Miller et al., 1987; Chunwang et al., 2004; Bartoš et al., 2012). Testosterone secretion in deer follows an annual cycle, stimulated by changes in day length, and is strongly associated with reproductive state (Bubenik et al., 1990; Stewart et al., 2018). This cycle gives rise to the annual cycle of antler development and casting, where testosterone remains low during the period of antler growth, increases during antler calcification prior to the breeding season, then dramatically decreases following the breeding season, which results in antler casting (Bubenik, 1982; Bubenik et al., 1975, 1982; DeYoung & Miller, 2011; Morris & Bubenik, 1982).

Although previous research has been foundational in establishing testosterone's association with day length, reproductive state, and the antler cycle, much of our knowledge of these patterns in deer has been generated using captive populations, where behaviors and interactions among individuals may differ from freely breeding populations (Bubenik & Leatherland, 1984; Killian et al., 2005; Mirarchi, Scanlon, Kirkpatrick, & Schreck, 1977; Rolf & Fischer, 1996; Stewart et al., 2018). As a result, our understanding of how testosterone influences ecological patterns in white‐tailed deer and other ungulates is limited. Furthermore, while testosterone trends have been described at the population level, studies focusing on how individuals may differ within age classes and in lifetime patterns are limited (Bubenik & Schams, 1986; Ditchkoff et al., 2001; Mirarchi, Mirarchi, Scanlon & Kirkpatrick, 1977; Pavitt et al., 2015). Evaluating these lifetime differences between individuals may provide insight into the role testosterone plays in sexual selection and lifetime reproductive strategies (Hau, 2007; Williams, 2008). For example, this relationship may manifest through the relationship between early‐life testosterone and maximum testosterone. Since many characteristics in cervids can be limited by poor development early in life (Harmel, 1982; Harmel et al., 1989; Pavitt et al., 2014), it is possible that testosterone concentrations during early reproductive years may also relate to testosterone secretion and reproductive effort later in life. This becomes increasingly important when evaluating testosterone levels as they relate to sexual selection, as variation among individuals must exist for sexual selection to occur (Darwin, 1871). Because of the influence of testosterone on sexually selected traits, we expected significant differences among individuals, and that patterns of secretion may vary throughout life.

White‐tailed deer are an excellent model to examine these issues due to their competitive breeding system contingent on sexual selection, extensive research on the annual physiological patterns of this species, and well‐documented management history. White‐tailed deer have a polygynous breeding system, where sexually mature males increase their reproductive output through a combination of bigger antlers, bigger bodies, and increased age. Older herd age structure and balanced sex ratios promote a competitive environment, whereby reproductive success is skewed toward fewer individuals when compared to populations with female‐biased sex ratios and a young buck age structure (Newbolt et al., 2017). This research sought to characterize seasonal and lifetime patterns of testosterone production in sexually mature male white‐tailed deer in a freely breeding, enclosed population exhibiting the seasonally late reproductive season seen in much of the southeastern United States (DeYoung et al.,^2003^, ^2009^; Newbolt et al., 2017; Turner et al., 2019). Specifically, our objectives were to describe seasonal, age‐specific, and lifetime patterns of testosterone through annual sampling of individually identified, known‐age white‐tailed deer. Because our population, like others in the region, exhibited peak breeding in mid‐January, nearly two months later than most populations in temperate North America (Newbolt et al., 2017), we predicted testosterone concentrations would also peak later than in early‐breeding populations. Furthermore, we expected to see a positive relationship between testosterone and age and sought to investigate whether those differences exist throughout the year (Bubenik & Schams, 1986; Ditchkoff, Spicer, et al., 2001). We also expected to see differences among individuals in the population throughout their lives, since testosterone influences traits under sexual selection (Darwin, 1871; Jašarević et al., 2012). By establishing a foundational knowledge of patterns of testosterone secretion, we can further investigate the role that physiology plays in the lifetime behavioral and reproductive ecology of white‐tailed deer and other mammals.

## 2 MATERIALS AND METHODS

### 2.1 Study area

Data were collected at the Auburn Captive Facility (ACF), which was a part of Auburn University's Piedmont Agricultural Research Station in Camp Hill, Alabama. The facility maintained a population of 100–120 white‐tailed deer within a 174‐ha enclosure surrounded by a 2.6‐m fence. The population consisted of wild deer that were present in the area when the fence was erected in 2007, and their subsequent offspring. No outside deer were introduced into the population, and deer within the fence were not subject to hunting. The population was regulated through natural mortality, excluding predation, and selective release of fawns outside the facility. Previous research has shown that the peak of the breeding season, as determined by fetal aging date, is mid‐January (Newbolt et al., 2017).

Our population of white‐tailed deer simulated the breeding behaviors of a wild population, as individuals were freely breeding and wild‐behaving. However, the potential influences of nutritional limitations often present in wild populations (Bartoš et al., 2010; Fattorini et al., 2018) were minimized by providing year‐round ad libitum supplemental feed. The facility consisted of 40% open fields and 60% mixed forest. The forested areas had a closed canopy with little understory growth. Primary tree species found within the forest included oak (*Quercus* spp.), hickory (*Carya* spp.), maple (*Acer* spp.), and pine (*Pinus* spp.) of varying age classes. Bermuda grass (*Cynodon* spp.) was the most prevalent grass species in the fields, but fescue (*Festuca* spp.), big bluestem (*Andropogon gerardii*), Johnson grass (*Sorghum halepense*), dallisgrass (*Paspalum dilatatum*), and bahia grass (*Paspalum notatum*) were also common. Food plots were also present within the enclosure and contained various warm and cool season forages to provide supplemental nutrition (Waer et al., 1992). Additionally, three feeders containing 18% protein pellets (“Deer Feed,” SouthFresh Feeds, Demopolis, Alabama; Record Rack^®^, Nutrena Feeds, Minneapolis, MN) were available to deer ad libitum throughout the year to supplement nutrition. To attract deer for capture‐related purposes during fall and winter, four timed‐released feeders deployed approximately 2 kg of corn (*Zea mays*) daily.

### 2.2 Field methods

We evaluated testosterone data for bucks ≥1.5 years old to examine trends in testosterone concentrations throughout the prebreeding, breeding, and postbreeding seasons, and used samples collected during September–March of 2007–2017. We captured and immobilized deer using a mixture of Telazol^®^ (Fort Dodge Animal Health, Fort Dodge, Iowa) and xylazine (Lloyd Laboratories, Shenandoah, Iowa) administered to the hindquarters with telemetry darts (2.0 cc, type C, Pneu‐Dart Inc., Williamsport, PA). We administered Telazol^®^ at a concentration of 125 mg/ml and a rate of approximately 2.2 mg/kg, while we administered xylazine at a concentration of 100mg/ml given at a rate of approximately 2.2 mg/kg. We loaded the immobilizing drug mixture into darts equipped with radio transmitters and fired using a 0.22 caliber blank (Kilpatrick et al., 1996). Using VHF telemetry, we located immobilized deer. If necessary, deer resistant to the tranquilizer mixture received additional mixture. After deer were moved to the necessary location for data collection and data collection was complete, we injected Tolazoline (1.5 ml/45.36 kg) in equal amounts into muscle in the shoulder and hindquarters to reverse sedation.

Upon initial capture, we aged individuals using tooth replacement and wear, then assigned a unique 3‐digit individual identification number visibly displayed on ear tags (Newbolt et al., 2017; Servinghaus & Moen, 1985). We collected 10 ml of blood for testosterone analysis via venipuncture of the jugular vein, centrifuged the samples to separate blood cells from serum, and stored them at −80°C in Cryule plastic cryogenic vials (Wheaton, Millville, NJ). All animal handling and research in this study were approved by the Auburn University Institutional Animal Care and Use Committee (PRN 2008‐1421; PRN 2010‐1785; PRN 2013‐2372; PRN 2016‐2964; PRN 2019‐3599).

### 2.3 Testosterone measurement

We analyzed serum testosterone concentrations using enzyme‐linked immunosorbent assays (ELISAs; Gionfriddo et al., 2011). Prior to ELISAs, we performed hormone extraction based of the Steroid Liquid Sample Extraction Protocol from Arbor Assays Inc. (Ann Arbor, MI), with modifications made for available equipment and optimal sample concentrations, done by adding 1 ml ethyl acetate to 0.1 ml serum and vortexing the mixture. The mixture was then frozen at −20°C. We poured the top solvent layer off, then performed extraction once more, repeating the procedure. We then dried samples in glass test tubes via a hot water bath at 60–65°C for 12–24 hr, then cooled samples to room temperature in a fume hood. If samples were not assayed immediately after cooling, they were covered, sealed, and stored at −20°C until assays could be completed.

To prepare extracted samples for ELISA assays, we dissolved samples at room temperature to a concentration of 0.8 µl using 250 µl DetectX^®^ Testosterone Enzyme Immunoassay Kit Assay Buffer. We ran samples in duplicates using the procedures and materials provided in the DetectX^®^ Testosterone Enzyme Immunoassay Kit (#K032‐H5) from Arbor Assays Inc., Ann Arbor, MI. Cross reactivity with other hormones for this kit reported by the manufacturer was as follows: testosterone 100%, 5a‐dihydrotestosterone 56.8%, androstenedione 0.27%, androsterone 0.04%, DHEA 0.04%, cholesterol 0.03%, 17b‐estradiol 0.02%, progesterone < 0.02%, pregnenolone < 0.02%, hydrocortisone < 0.02%, and cholic acid and derivatives < 0.02%. This kit reports a sensitivity of 9.92 pg/ml and limit of detection of 30.6 pg/ml. We read optical density from samples following the addition of the colorimetric substrate at 450nm using a Molecular Devices Spectra Max 190 plate reader and Molecular Devices SoftMax^®^ Pro (Copyright ^©^ 1999–2009 MDS Analytical Technologies, US, Inc.) software. Using “Arbor Assays Testosterone EIA kit” online data analysis tool, (MyAssays Ltd., accessed 9 January 2019 through 21 August 2019, at https://www.myassays.com/arbor‐assays‐testostrone‐eia‐kit.assay), we calculated concentrations of testosterone from absorbance data. To do this, we compared absorbance of sample‐filled wells to that of standardized samples prepared to concentrations of 10,000, 4,000, 1,600, 640, 256, 102.4, and 40.96 pg/ml. From the absorbance reading of these known standardized samples, we created a standardized curve. To calculate testosterone concentration of serum samples, we compared absorbance of serum to the absorbance of the standardized curve.

### 2.4 Statistical analysis

We calculated mean monthly testosterone, by age class, with bucks 1.5–2.5 years old classified as “immature” and bucks ≥ 3.5 classified as “mature.” Although all bucks of both age classes are sexually mature, male white‐tailed deer may not reach physical maturity until around age 3 (Michel et al., 2018). We use the term “immature” to refer to these sexually mature, but physically not fully developed ages. Both age classes have been documented to be reproductively active and capable of siring offspring in this population (Neuman et al., 2016; Newbolt et al., 2017). Our approach was consistent with age classifications used in previous research and the typical lifespan of wild deer in a hunted population (Michel et al., 2018; Strickland & Demarais, 2000). We assessed differences between average testosterone of these age groups for each month of our study using two‐sample *t* tests. Furthermore, we evaluated the relative strength of support for models of the relationship between testosterone concentration and age fitted as a quadratic continuous variable, month, individual, and an interaction term between age and month, using Akaike's Informational Criteria (AIC). We considered models with ∆AIC*_c_* values < 2 as informative (Arnold, 2010). All statistical analyses were performed in the R software v3.6.1 (R Core Team, 2014).

To analyze lifetime patterns of testosterone for individuals, we created a model that captures variation in testosterone with respect to age, month, and year. Because we did not sample every individual at every age, and across all temporal scales, this method allowed us to better compare testosterone level while accounting for variables that impact testosterone concentration. To do this, we ran a linear mixed effects model with fixed effects for age, month sampled, an interaction between age and month, and a random effect for individual and year sampled. In this analysis, age was treated as a continuous integer, rather than grouped into the aforementioned age categories. To assess the differences among individuals within our population, we used this same model to generate testosterone values for each individual that accounted for the effects of capture date, age of the individual, and random effects for individual and capture year. Using predictions from this model, we generated these corrected testosterone levels, which accounted for differences in testosterone that may result as an artifact of sampling month, sampling year, and age of the individual.

To compare yearling testosterone to maximum testosterone, we subset our data to include only individuals that we had sampled at age 1.5, and at ≥1 other point in their lives. If individuals had been sampled more than once after age 1.5, we utilized the maximum corrected testosterone level, generated from the predictions from the aforementioned top model for our analyses. We then used a linear mixed effects model with a random effect for individual to assess the relationship between yearling corrected testosterone level and the maximum corrected testosterone level that an individual had over the course of the study period. We wanted to assess lifetime patterns of testosterone for each individual, to see if range in testosterone over the course of their lives related to their average corrected testosterone level. Because of testosterone's close relationship to reproductive efforts, comparing these values may provide insight into differences in lifetime reproductive strategies. To do this, we subset our original data to include individuals captured ≥2 times at any point throughout the capture period, regardless of age at capture. Using age‐ and month‐corrected testosterone values, we calculated each individual's range of corrected testosterone levels, and mean corrected testosterone levels. We used a generalized linear model with a Gaussian family, for continuous decimal data with a normal distribution, to assess the relationship between testosterone range and mean testosterone.

## 3 RESULTS

**FIGURE 1:**
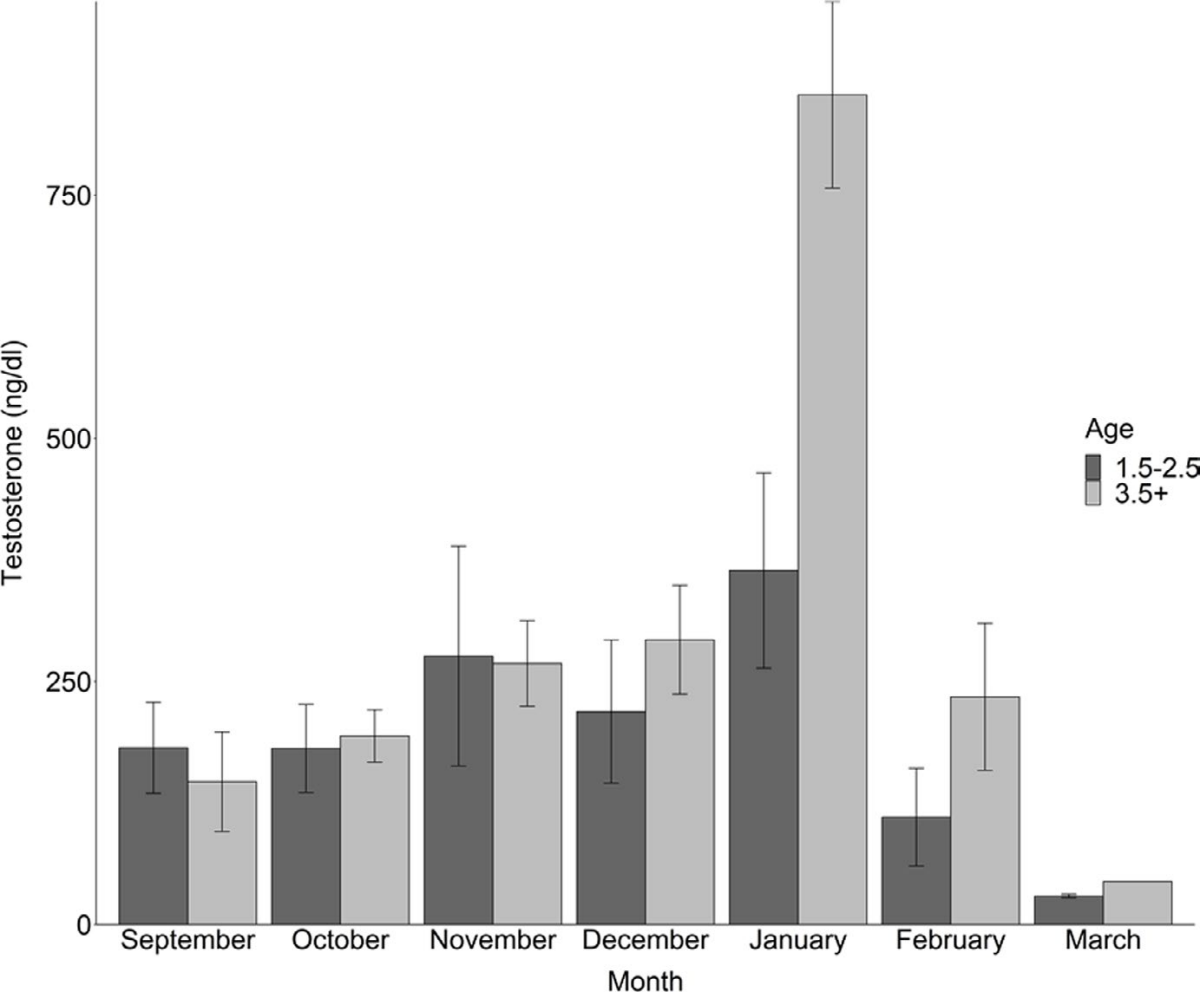
Monthly average testosterone concentrations of male white‐tailed deer captured at the Auburn Captive Facility, September–March, 2007–2017. Testosterone concentrations were greater for all males during the breeding season (January), but greater for mature than immature males

In total, we sampled 88 individual deer. On average, individuals were captured 2.44 times throughout the course of the study. The most a single individual was sampled was seven times, which occurred for three individuals. 36 individuals were sampled once and 52 were sampled at least twice within the study period. Of the individuals sampled at least twice, 22 individuals were sampled as yearlings and at one other age. We measured testosterone of 151 samples from bucks aged 1.5–2.5 years old and 77 samples from bucks aged ≥3.5 years old (*n* = 228) from September–March, 2007–2017. Population monitoring efforts, as described in Newbolt et al., (2017), indicated that >90% of the adult deer population was captured during the study period. Testosterone concentrations ranged from 1.12 to 2,432 ng/dl. The best fit (and only competitive) model for serum testosterone included age, month, and the interaction between those factors (Table 1). This model also had the greatest weight (>0.9). Our linear mixed effect model showed that month, age, and the age*month interaction were related to testosterone concentration (Table 2). Furthermore, a standard deviation of ±63.38 ng/dl for our random effect for individual informed us that testosterone may vary by up to 126.76 ng/dl due to differences between individuals. In general, testosterone increased from September to January, where it peaked, but was greater for mature bucks (853.17 ng/dl ± 95.88 *SE*) than immature bucks (364.05 ng/dl ± 100.37 *SE*; *p* = 0.012; Figure 1) during January. Outside of the breeding season, we found no significant age‐related differences between immature (183.82 ng/dl ± 30.29 *SE*) and mature bucks (221.30 ng/dl ± 22.16 *SE*; *p* > 0.136).

**TABLE 1:** AIC model selection for factors that influence testosterone concentration for male white‐tailed deer captured at the Auburn Captive Facility, September—March 2007—2017

Model	*K*	∆AIC*_c_*	*w_i_*
Age^2^ + Month + Age^2^ × Month + R	24	0	1
Age^2^ + Month + R	12	218.16	0.00
Month + R	10	257.35	0.00
Age^2^ + R	6	330.85	0.00
1 + R (Null)	4	368.69	0.00

Note

Factors in candidate models included: quadratic effects of age (Age^2^), month during the sampling period, an interactive effect between age^2^ and month, and random effects for individual male and sample year (R).

**TABLE 2:** Table of coefficients for our top model for factors that influence testosterone concentrations for male white‐tailed deer at the Auburn Captive Facility, September–March 2007–2017

Parameter	Estimate	*SE*	*df*	*p*
January	628.71	51.11	64.30	<0.001
February	−461.68	73.46	191.88	<0.001
March	−556.07	231.89	192.67	0.02
September	−543.71	261.71	193.44	0.04
October	457.56	60.83	175.13	<0.001
November	−293.44	71.52	186.06	<0.001
December	−36.08	101.15	191.55	0.72
January: Age	4,440.59	703.88	192.64	<0.001
February: Age	−3,452.90	1,289.99	190.62	0.01
March: Age	−3,179.79	8,059.83	192.43	0.69
September: Age	−5,889.46	6,536.06	191.23	0.37
October: Age	−4,082.19	838.78	192.16	<0.001
November: Age	−4,616.99	1,041.73	193.54	<0.001
December: Age	5,681.13	2,304.26	191.99	0.01
January: Age^2^	−796.50	629.07	192.29	0.21
February: Age^2^	267.36	1,653.78	193.52	0.87
March: Age^2^	1,971.95	8,334.08	192.50	0.81
September: Age^2^	−1,306.41	6,781.17	190.06	0.85
October: Age^2^	1,309	765.77	193.65	0.09
November: Age^2^	−386.56	1,006.87	189.63	0.70
December: Age^2^	7,335.92	2,740.26	192.80	0.01
Random Effects	Var.	*SD*		
Individual	4,018	63.38		
Year	3,227	56.80		
Residual	84,283	290.31		

Note

Estimates are reflective of testosterone concentration relative to January for a deer age 0.

**FIGURE 2:**
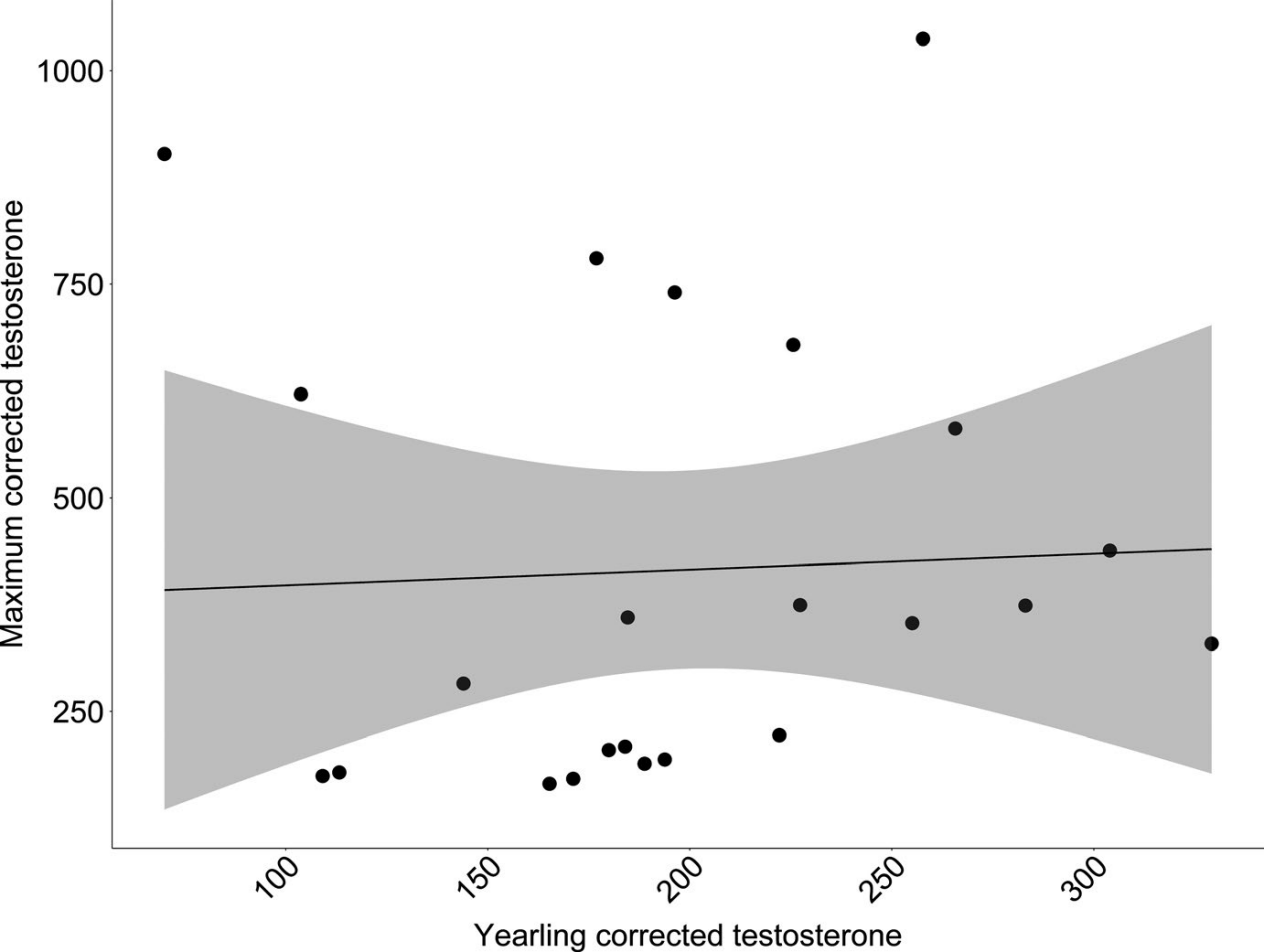
Yearling testosterone level compared to maximum testosterone level for individuals captured at the age of 1.5 years and at least one other time throughout the study period (2007–2017) at the Auburn Captive Facility, Auburn, AL. Testosterone concentrations were corrected to account for the effects of month, age, and a random effect of capture year

**FIGURE 3:**
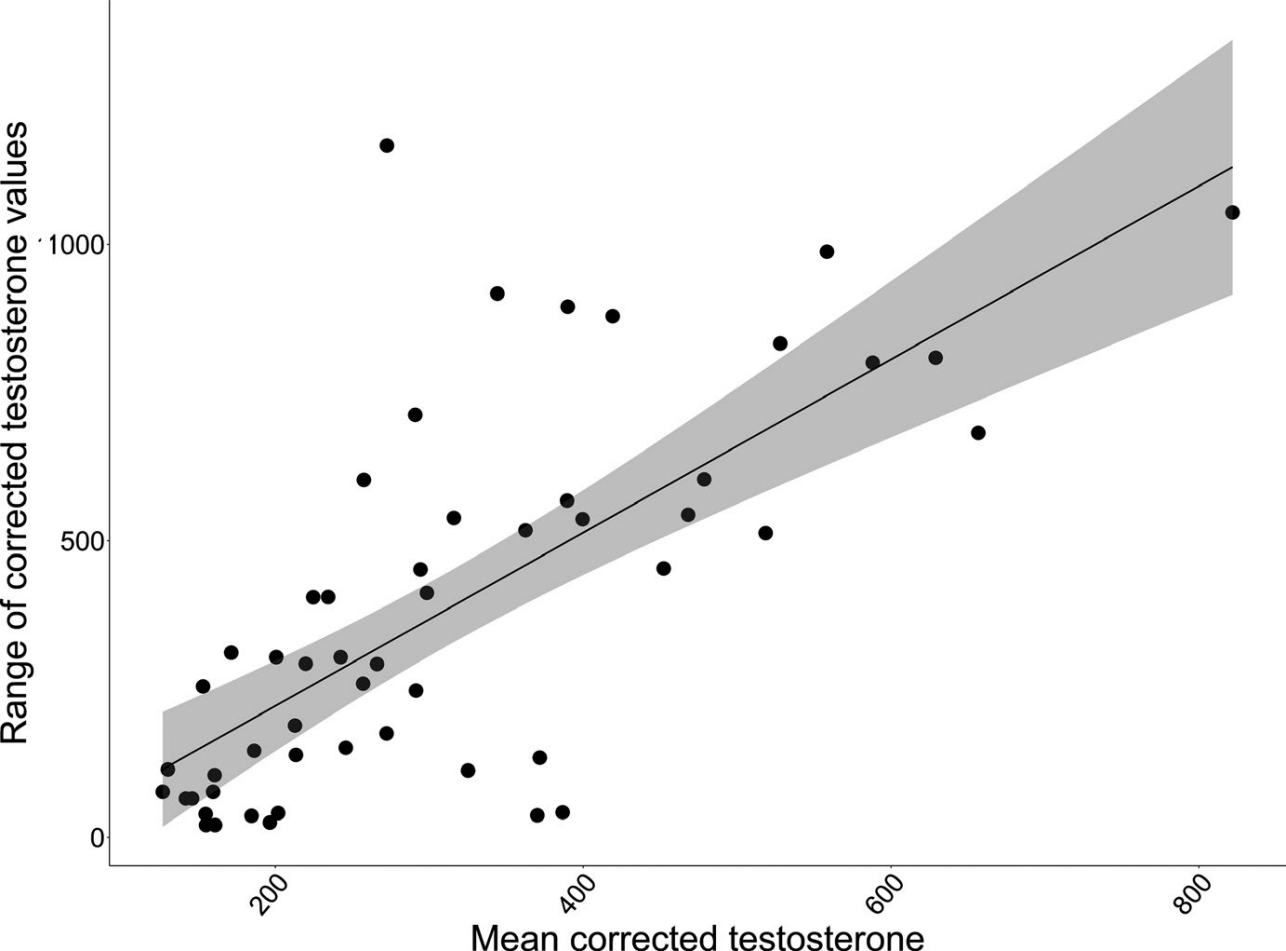
Individual average testosterone level related to range of lifetime testosterone level. Individuals included were those captured multiple times at the Auburn Captive Facility, Auburn, AL, over the course of the study (2007–2017). Testosterone concentrations were corrected for the effects of month, age, and a random effect of capture year greater mean testosterone for an individual was positively correlated with lifetime variation in testosterone

We compared corrected testosterone levels of 22 bucks captured at 1.5 years to the maximum corrected testosterone level measured over their lifetime. We found that an individual's corrected testosterone level at 1.5 years of age was not correlated with maximum corrected testosterone level later in life (*p* = 0.583; Figure 2). When comparing an individual's mean corrected testosterone level to the range of corrected testosterone levels produced over that individual's lifetime (testosterone variation), we found a positive association (*p* < 0.001) between mean corrected testosterone level and testosterone variation. For every 1 ng/dl increase in an individual's range of testosterone values, an individual's mean testosterone increased by 1.54 ng/dl (±0.233 *SE*; Figure 3).

## 4 DISCUSSION

We observed concentrations of testosterone near the range of 50–2,000 ng/dl previously reported for white‐tailed deer in other studies. However, the range of concentrations often differs between studies conducted on wild versus captive individuals, potentially due to differences in the degree of social interactions (Mirarchi, Scanlon, Kirkpatrick, & Schreck, 1977). Although these differences persist throughout the year, they are accentuated during the breeding season (Mirarchi et al., 1978). During the peak of the breeding season, when testosterone levels peak, captive deer have been reported to produce testosterone concentrations of 1,330–1,540 ng/dl (Bubenik & Bubenik, 1985; Mirarchi et al., 1978), compared to 2,370 ng/dl in wild deer (Mirarchi et al., 1978). Outside of the breeding season, wild deer have slightly greater testosterone; however, both wild and captive deer have testosterone concentrations less than 300 ng/dl (Mirarchi et al., 1978). The peak concentrations for our population reached 2,432 ng/dl during the peak of the breeding season, similar to the peak reported in wild populations (Mirarchi et al., 1978). We observed testosterone concentrations as low as 1.12 ng/dl outside of the breeding season, similar to the low values seen in captive herds (Bubenik & Bubenik, 1985; Stewart et al., 2018), and slightly less than what has been reported in wild populations (McMillin et al., 1974). However, it is worth noting that these measurements may also differ slightly due to differences between laboratories, assays, and sample preparation. These results, coupled with overwhelming support for our top model, suggest that time of year and age both play a role in determining testosterone concentration, and that the effect of age also varies depending on time of the year.

We observed the typical pattern of low testosterone during antler growth, greater concentrations leading up to peak breeding, and a dramatic decrease following the breeding season. While peak concentrations of testosterone in our study occurred during the month of January, 2–3 months later than other studies conducted on both captive and wild white‐tailed deer in temperate regions (McMillin et al., 1974; Mirarchi et al., 1978; Bubenik et al., 1983; Bubenik & Bubenik, 1985; Bubenik & Schams, 1986; Miller et al., 1987; Ditchkoff, Spicer, et al., 2001; Stewart et al.,. 2018), peak testosterone levels still coincided with the peak of conceptions in this population (Newbolt et al., 2017). This is consistent with previous data reported by Bubenik et al., (1990) that documented testosterone peaks associated with peak breeding season in both southern Texas (latitude 27°N) and southern Ontario (latitude 42°N). White‐tailed deer in Texas experienced both peak breeding and peak testosterone concentrations in December, 1 month later than deer in Ontario. Similarly, many populations in Alabama (Lueth, 1955) and neighboring southeastern states (Mississippi, Jacobson et al., 1979; and Louisiana, Roberson and Dennett, 1966), including our study area (latitude 33°N), exhibit a later breeding season than other temperate regions, occurring in January rather than October or November. It is hypothesized that differences in reproductive timing throughout the range of white‐tailed deer have evolved through genetic differences that exist with regard to photoperiod response (Bronson, 1988). The later breeding season that occurs throughout many regions of Alabama is likely a product of restocking efforts that occurred up until over 50 years ago from populations (i.e., southwestern Alabama) that historically exhibited later breeding (Lueth, 1967; Turner et al., 2019). It is hypothesized that this later breeding period may have been beneficial for deer populations in the Southeast to cope with local environmental pressures that may be present for an earlier fawning period, such as less forage availability and increased rainfall and flooding (Diefenbach & Shea, 2011). Following translocation, these animals maintained the reproductive chronology of their source populations, as the short, mild winters and long growing seasons common to the region likely did not subject the population to strong selection for earlier parturition dates. The differences in breeding timing that exist today are well‐documented through behavioral observations, later parturition timing, and genetic analyses (DeYoung et al., 2003; Newbolt et al., 2017; Turner et al., 2019). Marked genetic differences due to restocking are still apparent in wild populations today, despite a lack of geographical distance or barriers between restocked deer and native, earlier breeding individuals (DeYoung et al., 2003). Furthermore, deer with genetics from restocked populations tend to experience breeding timing that coincides largely with respective origin populations (Sumners et al., 2015), demonstrating the heritability of breeding chronology. Since testosterone cycles drive reproduction in males, it was unsurprising to find that the peak of the serum testosterone concentrations we observed coincided with the peak of the breeding season, despite a seasonally late breeding season (McCoy & Ditchkoff, 2012). Our data offer further evidence of the close relationship between reproductive chronology and testosterone concentrations for white‐tailed deer.

In our population, we observed a difference in the concise peak of testosterone correlating with a concise peak of the breeding season, rather than a prolonged period of elevated testosterone. While white‐tailed deer populations in other regions also exhibit relatively late breeding seasons, physiological patterns in these populations differ from our temperate‐region population. For example, in equatorial regions with later breeding seasons, testosterone concentrations and antler growth patterns are weakly associated with daylight shifts (McMillin et al., 1974). Furthermore, the breeding season in these regions is often prolonged in comparison to our study population and most temperate North American populations (Richter & Labisky, 1985). Essentially, the hormonal patterns we observed are most similar to those of other North American populations, with a concise breeding season and strong associations between decreases in day length, antler growth, and testosterone patterns (Newbolt et al., 2017), only occurring at a later time period. However, previous research by Turner et al., (2019) suggests that does from temperate populations experiencing later parturition may incur nutritional, developmental, and reproductive disadvantages entering their first breeding season as nutritional resources diminish. This differs from later, prolonged breeding seasons seen in more equatorial regions of white‐tailed deer, where nutritional resources remain available throughout the year, and deer do not incur as great a cost with late parturition. While other populations exhibit later breeding periods, the concise nature of the breeding season and concise peak in testosterone surrounding the breeding season in our study suggest that late‐breeding deer in temperate regions may still face some selective pressure resulting from seasonal limitations in nutrient availability in comparison to populations at tropical latitudes. Furthermore, this research highlights the long‐term implications that historical management practices may have on wildlife physiology.

Testosterone in our population generally increased with age, consistent with previous research in white‐tailed deer (Bubenik & Schams, 1986; Ditchkoff, Spicer, et al., 2001; Miller et al., 1987). In the context of the reproductive ecology of this species, increased testosterone at these ages correlates with greater reproductive investment. Ages at which deer have greater testosterone (Ages 3.5 up to 7 years; Bubenik & Schams, 1986; Ditchkoff, Spicer, et al., 2001) are also the ages typically associated with greater antler size (Hewitt et al., 2014), body size (Sauer, 1984; Strickland & Demarais, 2000), and reproductive output (Newbolt et al., 2017). However, bucks in these age classes also experience greater breeding season‐related mortality (Ditchkoff et al., 2001). Altogether, these findings confirm that testosterone is positively associated with the age classes at which deer invest more reproductive effort and incur the most breeding‐related stress.

While age‐related differences in testosterone occurred during the breeding season, they were not present throughout the remainder of our sampling period. A lack of age‐related differences outside of the breeding season suggests there is little benefit gained from maintaining elevated testosterone throughout the year. Although testosterone contributes to factors associated with reproductive success, testosterone‐mediated behavior during the breeding season imposes physical and immunological handicaps by increasing physical exertion and injury risk during fall and winter, when resources are limited (Folstad & Karter, 1992). Additionally, bucks often forgo nutritional resource acquisition to invest in intrasexual competition, mate chasing, copulation, and tending during the limited timeframe that does are in estrus (DeYoung & Miller, 2011; Ditchkoff, 2011). These activities often lead to injury or death during and after the breeding season (Ditchkoff, Welch, et al., 2001). Decreasing testosterone concentrations outside of the breeding season may serve as a compensatory trait, a mechanism evolved to cope with potential stressors imposed with sexually selected traits (Kirkpatrick, 1987). Given the potential physiological burdens of sustaining elevated testosterone, this pattern may indicate that elevated testosterone may only be beneficial in the context of preparation for and participation in the breeding season.

Our ability to obtain repeated samples from known individuals throughout their life allowed us to describe lifetime patterns of testosterone, something not often done in wild populations (Festa‐Bianchet, 2012). We found that an individual's corrected testosterone level as a yearling has no association with maximum testosterone later in life. Testosterone does not peak until later in life, and this later peak can be attributed to the Principle of Allocation (Levins, 1968). By this principle, younger individuals invest more heavily in somatic growth as opposed to reproductive effort. However, as deer age, and somatic growth consumes proportionally less energy, individuals may physiologically allocate more resources toward reproductive efforts. Increasing the age of peak reproduction and maturation may increase lifetime reproductive capacity. However, in species that experience maturation later in life, individuals face a greater risk of dying prior to peak reproductive age, negatively impacting lifetime reproductive success (Stearns, 1992). The greatest levels of testosterone and reproductive output occur later in life in deer. It is at these older ages that interindividual differences in reproductive effort are more pronounced. Consequently, yearling deer do not invest as heavily in reproduction as older deer, as indicated by smaller sexually selected characteristics (Hewitt et al., 2014; Sauer, 1984; Strickland & Demarais, 2000) and fewer offspring produced (Newbolt et al., 2017). Our findings support that investment in testosterone follows a similar pattern to these characteristics. Furthermore, this finding corroborates prior research on deer antlers that emphasizes uncertainty in estimating a male's physical potential from early‐life characteristics, and that variation may arise due to a combination of environmental effects and a life history that favors a delay in fully expressing genetic potential of traits important in reproductive success (Hewitt et al., 2014). The relative plasticity hypothesis, a derivative of the organization‐activation model, describes how wide ranges of intrasexual phenotypic variation may arise at different points in life for individuals (Moore et al., 1991). Our lack of an association between yearling testosterone and maximum testosterone suggests plasticity of hormone levels as adults. Although we were unable to directly assess how this may relate to lifespan in our population, as our study period only encompassed the lifespan of a few individuals, we believe future research should examine this relationship. Our observed relationship between yearling and maximum testosterone differs from trends seen in red deer (*Cervus elaphus*), where greater yearling antler size was positively associated with prime‐age body and antler size (Lemaître et al., 2018). Previous work assessing individual differences as ungulates age has focused on traits such as neonatal mass compared to juvenile survival or mass, often not extending much later in life (Festa‐Bianchet et al., 1996; Jorgenson et al., 1993; Sæther & Heim, 1993). Although previous work has shown that neonatal testosterone in red deer positively correlates with yearling survival (Pavitt et al., 2014), to our knowledge, our study is one of the first to assess how testosterone early in life might relate to individual testosterone later in life.

We also found that differences in lifetime patterns of testosterone exist between individuals. Individuals with low mean testosterone exhibit little testosterone variation throughout life, whereas others with greater mean lifelong testosterone exhibit greater testosterone variation throughout life. Such differences may influence lifetime reproductive efforts and reproductive success (Martin et al., 2013). These results differ from those for red deer, where differences in timing of reproductive senescence exist among individuals (Nussey et al., 2009), but not in testosterone levels (Pavitt et al., 2015). The variation in testosterone patterns that we observed is consistent with patterns of variation in sexually selected traits (Darwin, 1871; Jašarević et al., 2012). The premise of sexual selection is contingent on significant differences existing between individuals, often through traits that indicate quality of an individual. It follows that testosterone, which is affected by an individual's condition (Pérez‐Rodríguez et al., 2006) and influences many facets of breeding (Gomes & VanDenmark, 1974), differs among individuals within a species (Kempenaers et al., 2008).

In our population, individuals of different quality may seek to maximize reproductive effort through different reproductive strategies (Kokko, 1998) in the form of different lifetime testosterone patterns. Two competing lifetime reproductive strategies observed in long‐lived species like white‐tailed deer are often referred to as “live fast, die young” and “slow and steady” (Bonduriansky et al., 2008). The individuals in our population that consistently produce lower testosterone levels over their whole lives might exhibit tendencies of the “slow and steady” strategy. When sexually selected traits develop with age, as is the case with antlers in white‐tailed deer (Hewitt et al., 2014), sexual selection may favor a strategy that prolongs the lifespan of an individual. On the contrary, those individuals exhibiting a wide range of testosterone and greater overall average testosterone level might exhibit a “live fast, die young” reproductive strategy, where individuals exhibit a shorter, but more physically stressful breeding lifespan (Lemaître et al., 2018; Rolff, 2002). Individual differences in body growth rate early in life have been documented for white‐tailed deer, and it is hypothesized that these different growth rates may influence lifetime reproductive success (Michel et al., 2018). However, the limited number of mortalities that have occurred within our study population prevents us from determining if there is a relationship between testosterone patterns and mortality rates. In the future, relating lifetime testosterone patterns to factors such as lifespan and senescence would prove helpful in understanding the role lifetime testosterone patterns may play in life history strategy. Based upon our data and previous research, we believe that further exploration of lifetime reproductive strategies in white‐tailed deer should be investigated, and that including hormonal patterns may provide insight into such patterns.

## CONFLICT OF INTEREST

None declared.

## AUTHOR CONTRIBUTION

**Monet Arianna Gomes:** Conceptualization (equal); Data curation (supporting); Formal analysis (lead); Methodology (supporting); Project administration (supporting); Resources (equal); Writing‐original draft (lead); Writing‐review & editing (equal). **Stephen S. Ditchkoff:** Conceptualization (equal); Funding acquisition (lead); Methodology (lead); Project administration (lead); Resources (equal); Supervision (lead); Writing‐review & editing (equal). **Sarah Zohdy:** Methodology (supporting); Resources (equal); Writing‐review & editing (equal). **William D. Gulsby:** Writing‐review & editing (equal). **Chad H. Newbolt:** Data curation (lead); Funding acquisition (supporting); Methodology (supporting); Project administration (supporting); Resources (equal); Writing‐review & editing (equal).

## Data Availability

Data are available from Dryad. https://doi.org/10.5061/dryad.2ngf1vhn3.
